# Effect of pharmacogenetics on plasma lumefantrine pharmacokinetics and malaria treatment outcome in pregnant women

**DOI:** 10.1186/s12936-017-1914-9

**Published:** 2017-07-03

**Authors:** Ritah F. Mutagonda, Appolinary A. R. Kamuhabwa, Omary M. S. Minzi, Siriel N. Massawe, Muhammad Asghar, Manijeh V. Homann, Anna Färnert, Eleni Aklillu

**Affiliations:** 1 Department of Clinical Pharmacy and Pharmacology, School of Pharmacy, Muhimbili University of Health and Allied Sciences, P.O. Box 65013, Dar es Salaam, Tanzania; 2 Department of Obstetrics and Gynaecology, School of Medicine, Muhimbili University of Allied Sciences, P.O Box 65013, Dar es Salaam, Tanzania; 3 Unit of Infectious Diseases, Department of Medicine, Karolinska Institutet, Solna, 171 76 Stockholm, Sweden; 4 Department of Infectious Diseases, Karolinska University Hospital, Stockholm, Sweden; 5 Section of Pharmacogenetics, Department of Physiology and Pharmacology, Karolinska Institutet, 171 77 Stockholm, Sweden

**Keywords:** Malaria in pregnancy, Day 7 lumefantrine concentration, Recrudescence, Pharmacogenetics

## Abstract

**Background:**

Pregnancy has considerable effects on the pharmacokinetic properties of drugs used to treat uncomplicated *Plasmodium falciparum* malaria. The role of pharmacogenetic variation on anti-malarial drug disposition and efficacy during pregnancy is not well investigated. The study aimed to examine the effect of pharmacogenetics on lumefantrine (LF) pharmacokinetics and treatment outcome in pregnant women.

**Methods:**

Pregnant women with uncomplicated falciparum malaria were enrolled and treated with artemether-lumefantrine (ALu) at Mkuranga and Kisarawe district hospitals in Coast Region of Tanzania. Day-7 LF plasma concentration and genotyping for*CYP2B6 (c.516G*>*T, c.983T*>C), CYP3A4*1B, CYP3A5 (*3, *6, *7) and *ABCB1 c.4036A4G* were determined. Blood smear for parasite quantification by microscopy, and dried blood spot for parasite screening and genotyping using qPCR and nested PCR were collected at enrolment up to day 28 to differentiate between reinfection from recrudescence. Treatment response was recorded following the WHO protocol.

**Results:**

In total, 92 pregnant women in their second and third trimester were included in the study and 424 samples were screened for presence of *P. falciparum*. Parasites were detected during the follow up period in 11 (12%) women between day 7 and 28 after treatment and PCR genotyping confirmed recrudescent infection in 7 (63.3%) women. The remaining four (36.4%) pregnant women had reinfection: one on day 14 and three on day 28. The overall PCR-corrected treatment failure rate was 9.0% (95% CI 4.4–17.4). Day 7 LF concentration was not significantly influenced by *CYP2B6*, CYP3A4*1B and *ABCB1 c.4036A*>*G* genotypes. Significant associations between *CYP3A5* genotype and day 7 plasma LF concentrations was found, being higher in carriers of *CYP3A5* defective variant alleles than CYP3A5*1/*1 genotype. No significant influence of *CYP2B6*, *CYP3A5* and *ABCB1 c.4036A*>*G*enotypes on malaria treatment outcome were observed. However, CYP3A4*1B did affect malaria treatment outcome in pregnant women followed up for 28 days (P = 0.018).

**Conclusions:**

Genetic variations in *CYP3A4* and *CYP3A5*may influence LF pharmacokinetics and treatment outcome in pregnant women.

## Background

Pregnancy-induced physiological changes alter the pharmacokinetic properties of a number of anti-malarial drugs, usually resulting in lower drug exposures and lower cure rates especially in advanced pregnancy stage compared to non-pregnant population [1, 2]. Pregnancy-associated physiological changes result in lower drug absorption, enhances drug clearance, and increase body fluid volume of distribution [3–5]. Drug exposure depends greatly on the rate of metabolism and differences in activity of metabolizing enzymes can significantly alter the efficacy of drugs. Elevated levels of oestrogens, progesterone, cortisol, and prolactin during pregnancy have been linked to altered expression and metabolic activity of several hepatic cytochrome P450 enzymes. For instance, catalytic activity of CYP3A4, CYP2C9 and CYP2A6 enzymes increases during pregnancy, while CYP2C19 and CYP1A2 enzyme activity decreases [6, 7]. These enzymes are involved in metabolism of several anti-malarial drugs including LF and artemether [8–10], and are genetically polymorphic displaying wide inter-individual variations in enzyme activity [11–16].

Lower drug exposure levels in pregnant women have been reported for artemether/dihydroartemisinin, artesunate/dihydroartemisinin, dihydroartemisinin and lumefantrine (LF) [1, 17–19]. This increases the risks of treatment failure, increase risk of adverse outcomes for the fetus associated with malaria complications, and development of resistance to malaria parasites. A higher treatment failure rate has indeed been observed in pregnant women compared to non-pregnant ones living in the same area [1, 19, 20]. In this case, treatment failure may not be caused by intrinsic parasite resistance but is instead the result of inadequate drug levels due to pregnancy, pharmacogenetic profile of the host or other non-genetic modifiers of the pharmacokinetic parameters.

Genetic variation in drug metabolizing enzymes and transporter proteins might predict plasma exposure and treatment failure and/or emergence of drug resistant pathogens on infectious and non-infectious diseases, such as malaria, HIV and tuberculosis [20–24]. CYP3A responsible for metabolism of artemether and LF is induced by approximately twofold during the third trimester of human pregnancy. Low cure rates (83.5%) have been reported for pregnant women in Thailand receiving ALu [2]. However, pregnant women in Uganda showed an adequate clinical response (98.2%) using similar doses of ALu [25]. The differences in these studies might be explained by differences in host genetics, pharmacokinetics, different resistance patterns of malaria parasites, or higher levels of background immunity among individual pregnant women [25]. Another study reported that the wide difference in CYP3A4*1B allele frequency between the Tanzania and Cambodia populations presents a potential explanation for the lower efficacy of ALu in Cambodia and highlighted the importance of pharmacogenetic considerations in the decision-making process of first-line treatment policies for specific populations [8].

To date, there are limited studies that attempted to examine the role of pharmacogenetics on the pharmacokinetics of anti-malarial drugs and treatment outcomes in pregnant women. The aim of this study is to investigate the effects of pharmacogenetics on day 7 LF plasma concentrations and treatment outcome in pregnant patients treated with ALu in Tanzania. The findings might have implications for treatment policies of not only anti-malarial drugs, in particular the widely used artemisinin-based combinations, but also other drugs metabolized by these enzymes.

## Methods

### Study design and population

This was a one-arm prospective cohort study that included all pregnant women who gave consent to participate in the study when attending antenatal clinics at Kisarawe and Mkuranga district hospitals, northern Tanzania. Between May 2014 to April 2015, pregnant women attending the antenatal clinics (ANCs) were screened for malaria infection by using malaria rapid diagnostic test (MRDT). Pregnant women with uncomplicated *Plasmodium falciparum* infection and haemoglobin level of >8 g/d were enrolled. The study received ethics approval from the institutional review board of Muhimbili University of Health and Allied Sciences (MUHAS). Participants were informed about the aim of the study and gave written consent before participating in the study. To ensure confidentiality, women's identification numbers were used when labelling samples and during data entry into confidential report forms (CRF).

### Sample size

Considering anticipated population proportion (P) of clinical failures in pregnant women being 18% [19], with 95% confidence level and 10% precision, 92 malaria positive pregnant women were enrolled in this study.

### Treatment, clinical procedures and follow-up

The study participants received six doses of four tablets of ALu (Coartem^®^; Novartis Pharma AG, Basel, Switzerland) (20 mg artemether and 120 mg lumefantrine) over the course of 3 days at 0, 8, 24, 36, 48, and 60 h. For each patient, general physical examination was performed at enrollment (day 0) and on follow up visits on days 2, 7, 14, 21 and 28. Approximately 50 μl of blood was collected on filter paper (Whatman grade 3) for later PCR analysis. Each filter paper was dried and individually stored in a plastic bag and kept frozen at −80 °C. At enrollment day, 1 ml of whole blood was taken into an EDTA containing vacutainer tube and stored at −80 °C at MUHAS laboratory until further analysis. Additionally, 3 mls of venous blood were drawn from pregnant women in heparinized tubes on day 7 to determine plasma LF concentrations.

### *CYP3A4, CYP3A5, CYP2B6*, and *ABCB1* genotyping

Genomic DNA was isolated from peripheral leukocytes in whole blood samples using QIAamp DNA Midi Kit (Qiagen GmbH, Hilden, Germany) according to the manufacturer’s instructions. Genotyping for the common functional variant alleles for CYP2B6*6, CYP2B6*18, CYP3A4*1B, CYP3A5*3, CYP3A5*6, CYP3A5*7 and *ABCB1 c.4036A*>*G (rs3842)*, which have been reported to be relevant for artemether and LF disposition [26, 27] were done as described previously [12, 22]. In brief genotyping was performed using TaqMan drug metabolism genotyping assay reagents for allelic discrimination (Applied Biosystems Genotyping Assays) with the following ID numbers for each SNP: C__7817765_60 for CYP2B6*6 (*c.516G4T,* rs3745274), C__60732328_20 for CYP2B6*18 (*c.983T4C,* rs28399499), C__26201809_30 for CYP3A5*3 (*c.6986A4G,* rs776746), C__30203950_10 for CYP3A5*6 (g.14690G4A,rs10264272) and C__32287188_10 for CYP3A5*7 (g.27131_27132insT rs41303343) and C__11711730_20) for *ABCB1 c.4036A*>*G* (rs3842), and C__11711730_20 for CYP3A4*1B (−*392A*>*G,* rs2740574). Genotyping was carried out using Quant Studio 12 K Flex Real-Time PCR system (Life Technologies Holding, Singapore, Singapore). The final volume for each reaction was 10 μl, consisting of TaqMan fast advanced master mix (Applied Biosystems, Waltham, MA, USA), TaqMan 20X drug metabolism genotyping assays mix (Applied Biosystems) and genomic DNA. The PCR profile consisted of an initial step at 60 °C for 30 s, hold stage at 95 °C for 10 min and PCR stage for 40 cycles step 1 with 95 °C for 15 and step 2 with 60 °C for 1 min and after read stage with 60 °C for 30 s.

### Parasite detection and genotyping

Dried blood spots on filter papers obtained at enrolment (day 0) and on follow-up days (day 2, 7, 14, 21 and 28) were punched, and one circle 5 mm in diameter was used for DNA extraction using QIAamp DNA blood micro kit (Qiagen GmbH, Hilden, Germany) following the manufacturer’s recommendations. Detection of *Plasmodium* parasites was performed using a species-specific PCR targeting the ssRNA gene [28]. PCRs were carried out in duplicate in a 25-μl final volume containing 12.5 μl of Universal PCR Master Mix, 5 μl of DNA, forward and reverse primers at various concentrations, and *P. falciparum*, *Plasmodium malariae*, *Plasmodium vivax* and *Plasmodium ovale* probes at a final concentration of 100 nM. All reactions were run on an ABI Prism 7000 sequence detection system (Applied Biosystems) with the default settings; each sample was initially denatured at 95 °C for 10 min and cycled 40 times, with each cycle consisting of 95 °C for 15 s and 60 °C for 60 s. Each reaction plate included four positive controls for *P. falciparum*, *P. ovale*, *P. vivax*, and *P. malariae* and a negative control with molecular-grade water in place of DNA.

PCR genotyping to differentiate recurrent *P. falciparum* infections from reinfections were done according to World Health Organization (WHO) recommendations [29], by characterizing the length polymorphism of the merozoite surface protein 2 gene (*msp2*) in samples collected at day 0 and on the day recurrent parasitaemia was found. Recrudescence was determined when at least one *msp2* allele of the same allelic type and of identical base pair size was found in samples collected on day 0 and on the day of recurrent infection. A reinfection was defined as all alleles were of different length in the sample collected on day 0 and that collected on the day of recurrent infection.

### Quantification of LF plasma concentrations

Blood samples for the determination of LF plasma concentrations were collected on day 7 (corresponding to 168 h) following initiation of ALu treatment. Blood samples for quantification of LF levels were centrifuged and plasma was stored at −80 °C until analysis. Plasma LF concentration was analysed using a validated method of high performance liquid chromatography (HPLC) with ultraviolet detection at Sida/MUHAS bioanalytical laboratory in Dar-es-Salaam, Tanzania [30]. The coefficients of variation (CV %) during the analysis of LF were 8.4, 4.7 and 4.5% at 100, 1000, and 8000 ng/ml, respectively. The lower limit of quantification was 50 ng/ml.

### Data analysis

LF plasma concentration data were log transformed to achieve normality of data distribution. Median (interquartile range) was used to describe day-7 LF plasma concentrations. Comparison of day-7 median LF plasma concentrations in pregnant women between the different genotypes were carried out using Kruskal–Wallis one-way analysis of variance (ANOVA). X^2^ test was used to compare the observed and expected allele frequencies according to the Hardy–Weinberg equilibrium. Influence of human genotype on malaria treatment outcome was analysed using Pearson’s Chi square and Fisher’s exact test. Haploview software package (version 4.2) was used to analyse linkage disequilibrium (LD) and haplotype construction. Statistical analyses were performed using Statistical Package for Social Sciences (SPSS) software, version 22.0 (IBM Corporation, Somers, NY, USA). P values <0.05 were considered to be statistically significant.

Malaria treatment outcomes were classified following the WHO protocol [31], as adequate clinical and parasitological response (ACPR), corrected for reinfection using PCR genotyping on day 28 and treatment failure (TF); designated as early treatment failure (ETF), late clinical failure (LCF), or late parasitological failure (LPF). The influence of genetic variations on malaria treatment outcome was evaluated using Cox regression analysis.

## Results

### Patient characteristics

In total, 1835 pregnant women were screened using MRDT and a total of 92 pregnant women with malaria infection consented and were enrolled in the study. Baseline characteristics are presented in Table 1. The median age of pregnant patients was 23 (range 15–41) years and the majority (54.3%) were multigravida. Most participants were in the second trimester (60.7%). The median parasite density was 2700 (range 400–72,500) parasites/µl.

**Table 1:** Baseline characteristics of study participants (n = 92)

Characteristic	Number of pregnant women	Percentage
Age (years)		
<18	8	8.7
19–25	52	56.5
>25	32	34.8
Gravida (n)		
Primigravida	42	45.7
Secundigravida	17	18.5
Multigravida	33	35.9
Trimester (n)		
Second	66	60.7
Third	26	39.3
Parasitaemia (parasites/µl blood)		
<1000	14	15.5
1000–10,000	51	55.0
>10,000	27	29.5

### Day 28 malaria treatment outcome in pregnant women

In total, 424 samples were screened for presence of parasites and *P. falciparum* was present in 11 (12%) patients during the follow up period. Two samples were detected on day 7, four on day 14, one on day 21 and four on day 28 after ALu treatment. PCR based genotyping of *msp2* confirmed recrudescent infection in seven women (63.3%), two on day 7, three on day 14, one on day 21 and one on day 28. The remaining four (36.4%) pregnant women had reinfection; one on day 14 and three on day 28. The overall rate of reinfection was 4.3% (95% CI 0.15–8.45).

PCR uncorrected ACPR on day 28 was 88.9% (95% CI 82.06–95.74) and TF rates were 11.1% (95% CI 4.26–17.94). Rate of ACPR was calculated using per-protocol method, where patients were excluded due to lost to follow-up, protocol violations and TF due to reinfection. PCR-corrected ACPR rate as defined by absence of parasitaemia on day 28, irrespective of axillary temperature, in patients who did not previously meet any of the criteria of ETF, LCF or LPF was 91.0% (95% CI 82.62–95.58). The rate of PCR-corrected LPF, as defined by presence of parasitaemia on any day between day 7 and 28 in patients who did not previously meet any of the criteria of ETF or LPF, was 9.0% (95% CI 4.42–17.38). There was no ETF or LCF.

### Allele, genotype and haplotype frequency distributions

The overall CYP2B6*6, CYP2B6*18, CYP3A4*1B, CYP3A5*3, CYP3A5*6, CYP3A5*7, *ABCB1 c.4036A*>*G* genotype and allele frequencies in Tanzanian pregnant women is presented in Table 2. There was no significant deviation between the observed and expected genotype frequencies from Hardy–Weinberg equilibrium. The variant allele frequency was highest (76.1%) for CYP3A4*1B followed by *CYP2B6 c.516G*>*T* (*6, 33.5%). *CYP2B6 c.983T*>*C* (*18) had the lowest allele frequency which was 9.3%. Genotype of CYP450 enzymes and ABCB1 transporter was equally distributed among the age, gravida, trimester and PMTCT status of pregnant women (P > 0.05).

**Table 2:** Genotype and variant allele frequency distribution among pregnant women with uncomplicated *Plasmodium falciparum* infection in Tanzania

Genotype	Frequency
	N (%)
*CYP2B6 c.516G*>T (*6)	
*1/*1	38 (46.3%)
*1/*6	33 (40.2%)
*6/*6	11 (13.4%)
*CYP2B6 c.983T*>C (*18)	
*1/*1	75 (82.4%)
*1/*18	15 (16.5%)
*18/*18	1 (1.1%)
CYP3A4*1B (−*392A*>*G)*	
*1/*1	3 (3.2%)
*1/*1B	38 (41.3%)
*1B/*1B	51 (55.4%)
CYP3A5*3 c.6986A>*G*	
*1/*1	51 (56.6%)
*1/*3	37 (41.1%)
*3/*3	2 (2.2%)
CYP3A5*6 c.14690G>*A*	
*1/*1	59 (64.1%)
*1/*6	28 (30.4%)
*6/*6	5 (5.4%)
CYP3A5*7 27131_27132insT	
*1/*1	68 (75.6%)
*1/*7	22 (24.4%)
*7/*7	0
*ABCB1 c.4036AG (rs3842)*	
AA	54 (58.7%)
AG	26 (28.3%)
GG	12 (13.4%)

Allele	Minor allele	%
*CYP2B6 c.516G*>T (*6)	*6	33.5
*CYP2B6 c.983T*>C (*18)	*18	9.3
CYP3A4*1B	*1B	76.1
CYP3A5*3	*3	22.8
CYP3A5*6	*6	20.6
CYP3A5*7	*7	12.2
*ABCB1 c.4036AG (rs3842)*	*G*	27.2

**Fig. 1:**
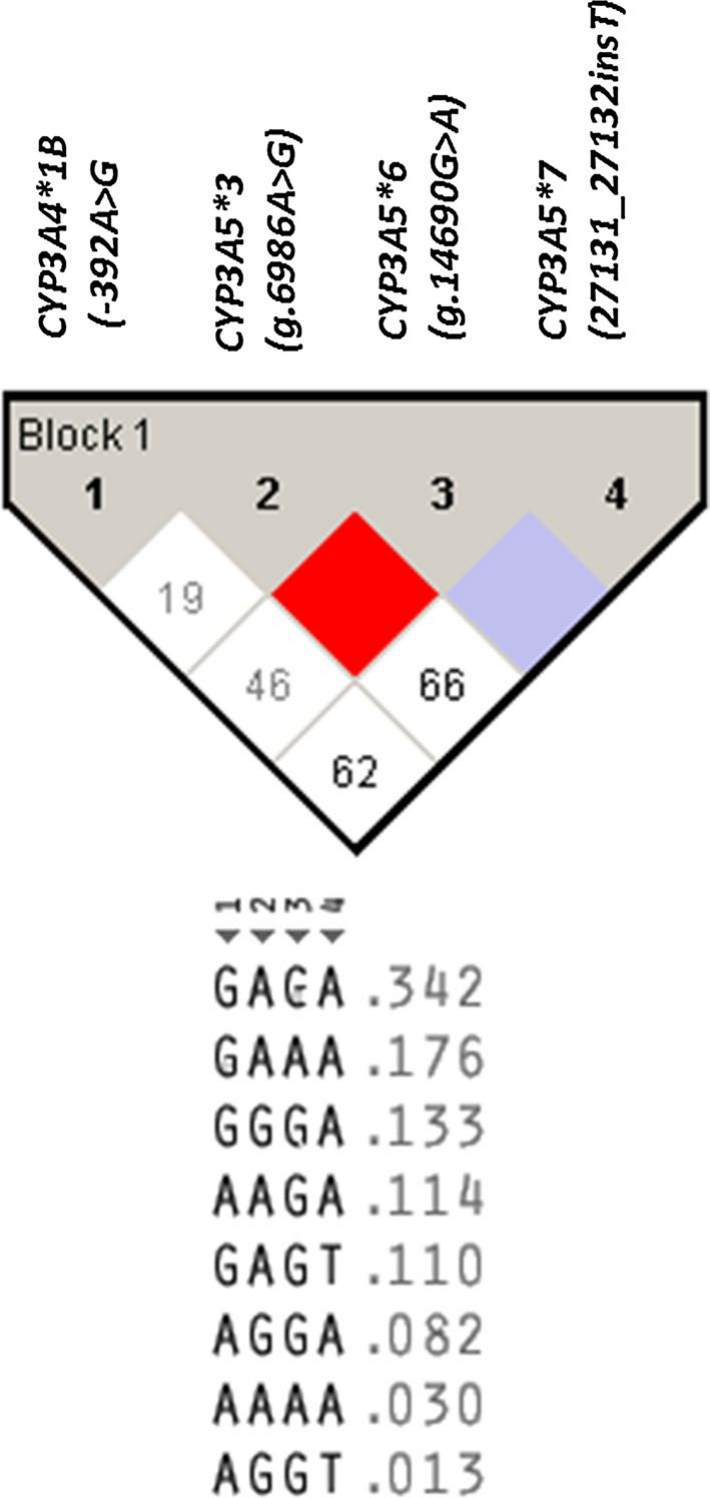
Linkage disequilibrium (LD) plot of CYP3A4*1B (−*392A*>*G)*, CYP3A5*3 (g.6986A>*G)*, CYP3A5*6 (g.14690G>*A)*, CYP3A5*7 (27131_27132insT) and the observed D′ values (within the *diagonal boxes*). The pair-wise LD relationship between two SNPs and the respective D′ value is indicated in each *square*. The color gradient from *red* to *white* reveals *higher* to *lower* LD (D′ 1–0) values

Haplotype analysis of CYP3A4*1B (–*392A*>*G)*, CYP3A5*3 (g.6986A>*G)*, CYP3A5*6 (g.14690G>*A)*, CYP3A5*7 (27131_27132insT) is presented in Fig. 1 and Table 3. There was no linkage between the two *CYP2B6* variant alleles (*c.516G*>*T* and *c.983T*>*C*). Likewise, no LD between the three *CYP3A5* SNPs (CYP3A5*3, CYP3A5*6, CYP3A5*7) was found. Instead each SNP was inversely linked to each other (i.e., each SNP is in strong LD with the wild type variant of the other two SNPs). The haplotype frequency of CYP3A5*1, *3, *6 and *7 was 44.5, 22.6, 20.7 and 12.2%, respectively. Interestingly all the three *CYP3A5* variant alleles (CYP3A5*3, CYP3A5*6 and *7) occur in high LD with CYP3A4*1B. The major *CYP3A* haplotype was CYP3A4*1B alone (34.2%) followed by its linkage with CYP3A5*6 (17.6%) and CYP3A5*3 (13.3%) (Table 3).

**Table 3:** CYP3A haplotype distribution among pregnant women with uncomplicated *Plasmodium falciparum* infection in Tanzania

CYP3Ahaplotype	*CYP3A* SNP combinations				Frequency (%)
	CYP3A4*1B −*392A*>*G*	CYP3A5 *3 *g.6986A*>*G*	CYP3A5 *6 *g.14690G*>*A*	CYP3A5 *7 *27131_27132insT*	
*GAGA*	*1B	wt	Wt	wt	34.2
*GAAA*	*1B	wt	*6	wt	17.6
*GGA*	1B	*3	Wt	wt	13.3
*AAGA*	wt	wt	Wt	wt	11.4
*GAGT*	*1B	wt	Wt	*7	11.0
*AGGA*	wt	*3	Wt	wt	8.2
*AAAA*	wt	wt	*6	wt	3.0
*AGGT*	wt	wt	Wt	*7	1.3

*wt* wild type variant allele, *SNP* single-nucleotide polymorphism

### The effect of genotype on day 7 LF concentration in pregnant women

Based on the haplotype analysis result, subjects were grouped according to the numbers of functional CYP2B6*1 and CYP3A5*1 variant alleles; two (*1/*1), one (heterozygous for defective variant alleles) and zero (homozygous for defective variant alleles) to investigate the effect of genotype on day 7 plasma LF concentrations.

The median day 7 plasma LF concentration was 650 with the range of 2936 (124–3059) ng/ml. Comparison of the median plasma LF concentrations between the different genotypes is presented in Table 4. There was no significant effect of *CYP2B6*, CYP3A4*1B or *ABCB1 c.4036AG* genotype on day 7 LF plasma concentrations. The median plasma LF concentration was significantly associated with *CYP3A5* genotypes (P = 0.039). *CYP3A5* defective allele carriers displayed a higher plasma LF concentrations compared to those homozygous for CYP3A5*1/*1 genotypes (P = 0.04). There was significant association between having *CYP3A5* genotype and having plasma LF concentration ≥600 ng/ml (Pearson Chi square test, P = 0.01). The proportion of subjects with two functional CYP3A5 genotype (*1/*1) was significantly higher among those with plasma LF conc <600 ng/ml (34.6%) compared to those with ≥600 ng/ml (5.9%). On the other hand proportion of subjects with lacking functional CYP3A5 (homozygous for defective variant alleles) was higher among those with plasma LF conc ≥600 ng/ml (38.2%) compared to those with >600 ng/ml (19.2%).

**Table 4:** Comparison of median and inter quartile range (IQR) of day 7 LF median plasma concentration between different genotype groups in pregnant women with uncomplicated *Plasmodium falciparum* infection using Kruskal–Wallis ANOVA

	Plasma lumefantrine concertation at day 7		
	n	Median (IQR)	*P* value
No of CYP2B6*1 allele			
Zero	17	650.0 (285.8–1603.2)	0.85
One	37	671.6 (280.0–1935.3)	
Two	28	672.0 (350.0–2161.8)	
CYP3A4*1B			
*1A/*1A	3	294.6 (270.0–319.2)	0.24
*1A/*1B	38	689.7 (354.1–1935.3)	
*1B/*1B	51	644.6 (280.0–2615.0)	
No of CYP3A5*1 allele			
Zero	27	716.6 (340.9–2109.4)	0.039
One	47	689.7 (280.0–3059.1)	
Two	18	354.2 (285.8–1603.2)	
*ABCB1 c.4036AG*			
AA	54	585.2 (330.0–2615.0)	0.28
AG	26	710.7 (282.3–2161.8)	
GG	12	1055.1 (285.8–2109.4)	

No of CYP2B6*1 allele: two, *1/*1; one, heterozygous for *6 or *18; zero, homozygous for *6 or *18 or combination thereof

No of CYP3A5*1 allele: two, *1/*1; one, heterozygous for *3, *6 or *7; zero, homozygous for *3, *6 or *7, combination thereof

### The effect of genotype on malaria treatment outcome in pregnant women

The possible influence of genetic variations in *CYP2B6, CYP3A5* and *ABCB1* on malaria treatment outcome was evaluated using Cox regression analysis. A log rank test was run to determine differences in the cumulative hazard distribution between the different genotypes. There were no associations between malaria treatment outcome and *ABCB1* transporter and most of CYP450 genotypes with an exception to *CYP3A4* (Table 5).

**Table 5:** Analysis of genetics effect on malaria treatment outcome (risk of recrudescence) in pregnant women

Genotype	No of patients	No. of recrudescence	Percentage (%)	P value (log-rank test)
CYP2B6*6				0.676
CYP2B6*1/*1	35	1	2.8	
CYP2B6*1/*6	32	2	6.5	
CYP2B6*6/*6	11	1	9.0	
CYP2B6*18				0.605
CYP2B6*1/*1	72	5	6.9	
CYP2B6*1/*18	14	2	14.2	
CYP2B6*18/*18	1	0	0	
CYP3A4*1B				0.018
CYP3A4*1/*1	2	1	50	
CYP3A4*1/*1B	37	3	8.1	
CYP3A4*1B/*1B	49	3	6.1	
CYP3A5*3				0.087
CYP3A5*1/*1	48	3	6.2	
CYP3A5*1/*3	36	3	8.3	
CYP3A5*3/*3	2	1	50	
CYP3A5*6				0.645
CYP3A5*1/*1	57	4	7.0	
CYP3A5*1/*6	26	3	11.5	
CYP3A5*6/*6	5	0	0	
CYP3A5*7				0.134
CYP3A5*1/*1	66	7	10.6	
CYP3A5*1/*7	20	0	0	
CYP3A5*7/*7	–	–	–	
*ABCB1 rs3842*				0.462
*A/A*	12	0	0	
*A/G*	25	3	12	
*G/G*	51	4	7.8	

## Discussion

In the present study, the effect of pharmacogenetics on LF pharmacokinetics (focusing on day 7 LF plasma concentration, which is a surrogate marker for AUC and malaria cure rate) and treatment outcome in pregnant women was investigated. For this purpose, functional variant alleles in CYP450 (*CYP2B6, CYP3A4, CYP3A5*) and *ABCB1* genes involved in the metabolism of anti-malarial drugs were assessed in pregnant malaria patients treated with ALu. The major finding includes: (i) *CYP3A5* genotype has significant influence on plasma LF concentration and (ii) CYP3A4*1B is associated with malaria treatment outcome. A number of studies have speculated that lower artemether and LF concentration in pregnant women has been due to changes in catalytic activities of CYP450 metabolizing enzymes particularly the increased activity of CYP3A4 [19, 20, 32]. This is the first study in pregnant women to investigate the effect of pharmacogenetics on day 7 LF concentrations and malaria treatment outcome after a follow up of 28 days. The observed variant allele frequencies for CYP2B6*6 (c.516G→T), CYP3A4*1B, and CYP3A5*3 are very similar to those previously reported from previous studies conducted in Tanzanian population [8, 12, 22, 23].

Lumefantrine, the long-acting component of the most widely used artemisinin-based combination therapy (ACT) in Africa is metabolized by CYP3A4. Artemether is metabolized by CYP3A4 and CYP3A5. Therefore, information on the role of pharmacogenetics on drug disposition and treatment outcome in pregnant women using ALu is needed. CYP3A5 is mainly expressed in black population and its genotype contributes to variations in the total CYP3A enzyme activity as measured by 4beta-hydroxycholesterol, an endogenous CYP3A marker [13, 15, 33, 34].

There was significantly higher plasma LF concentration in patients carrying *CYP3A5* defective alleles than those without. Though not significant, a similar finding was observed in a recent HIV-malaria cohort study in Tanzania [22]. Pregnant women withCYP3A5*1/*1 genotype had significantly higher risk of having LF plasma concentration <600 ng/ml. Plasma LF concentration <600 ng/ml is associated with risk of recurrent parasitaemia in pregnant women [20]. However, there was no significant impact of CYP3A5 genotype on malaria treatment outcome which is similar to previous reports [22]. This may indicate that CYP3A5 plays a minor role compared to CYP3A4 in determining malaria treatment outcome. Indeed, CYP3A4 is the major drug metabolizing enzyme than CYP3A5 for most CYP3A substrate drugs [35].

In this study, there was no influence of CYP3A4*1B on day 7 LF plasma levels. The findings are similar to previous reports from Cambodia and Tanzania [8]. A study conducted in Uganda reported that lower LF day 7 plasma concentrations observed during pregnancy were caused by a decrease of 36.5% in the inter-compartmental clearance in pregnant women [18]. The decrease in clearance can be attributed to changes in body composition (increased plasma volume, water, and fat content) during pregnancy [36] where the distribution of the lipophilic LF through the body might be altered substantially. In another study conducted in Tanzania it was also reported that CYP3A4*1B genotype did not affect day 7 LF plasma levels unless it was induced by a potent CYP3A4 inducer [22].

The study also analysed patients with recrudescence and compared their pharmacogenetic profiles with those with an ACPR. There was a significant association between CYP3A4*1B genotype and treatment outcome in pregnant women. It was observed that majority of pregnant women with CYP3A4*1B/*1B had attained ACPR compared to those with CYP3A4*1/*1. Inconclusive data on alterations of enzyme activity in CYP3A4*1B carriers are reported in the literature. Some investigators have suggested CYP3A4*1B is associated with increased CYP3A4 expression and enhanced drug elimination in carriers of CYP3A4*1B may lead to treatment failure [37]. In contrast, the association of CYP3A4*1B with lower CYP3A4 enzyme activity in Tanzanians is reported previously, where carriers of CYP3A4*1B had a significantly lower enzyme activity than CYP3A4*1 [38]. The finding of significantly lower total CYP3A activity in Tanzanians than whites (Swedes) and Asians (Koreans) despite having high allele frequency ofCYP3A4*1B in Tanzanians (77%) [34] may indicate the association of CYP3A4*1B with low enzyme activity in blacks. CYP3A4*1B variant allele is absent in Asians and occurs at a much lower frequency (2–9%) in whites [39].

Preliminary finding indicates that pharmacogenetic variation in *CYP3A4* and *CYP3A5* influences the LF plasma exposure and malaria treatment outcome in pregnant women. Since CYP3A is responsible for the metabolism of artemether and LF, interplay between *CYP3A4* and *CYP3A5* genotypes may determine ACT plasma exposure and treatment outcome. CYP3A4 and CYP3A5 haplotypes are located in the same gene locus, effects initially considered to be due to a CYP3A4 allele might actually be due to a CYP3A5 allele in LD [40]. LD between CYP3A4*1B and CYP3A5*1Ais suggested as possible cause of inter individual variation in CYP3A metabolism [41, 42].

Previous studies in white population reported that CYP3A4*1B is linked with the functional CYP3A5*1 resulting in high enzyme activity [42, 43]. In contrast our extensive haplotype analysis in Tanzanians (Fig. 1; Table 3) indicates that CYP3A4*1B is linked with *CYP3A5* defective variant alleles (*3, *5 and *7) and hence may be associated with low enzyme activity. It is well known that sub-Saharan African population is the most genetically heterogeneous population globally, characterized by extensive population substructure and unique LD pattern compared to non-African populations [44, 45]. Lack of LD between the two *CYP2B6* variant alleles (*c.516G*>*T* and *c.983T*>*C*) and between the three *CYP3A5* SNPs (*c.6986A4G*, *g.14690G4A* and *g.27131_27132 insT*) in this study is similar to previously reports from Africa [11, 13, 15, 23].

Limitation of this study includes that the sample size was calculated based on the previous study whereby treatment failure was reported to be 18% which is higher than the failure rate reported in this study. As a result, although the sample size is enough to investigate anti-malarial drug efficacy in the population, the study is under-powered to investigate the impact of *CYP3A* haplotypes on treatment outcome. However, the study finding highlights the importance of genetic variation in the *CYP3A* locus for LF pharmacokinetics and treatment outcome in pregnant women. This study also presents haplotype structure of the most common *CYP3A* functional variant alleles in African population for future genetic association studies.

## Conclusions

In general, the study finding indicates association of the low enzyme activity genotype of CYP3A4*5 and CYP3A4*1B with high LF plasma exposure and better malaria treatment outcome, respectively. The effects of pharmacogenetics on LF pharmacokinetics were well characterized in pregnant patients with uncomplicated *P. falciparum* malaria. The importance of these findings is that in the future genotyping can be used to predict the need for anti-malarial drugs dosage adjustment to pregnant women with CYP3A4*1/*1or CYP3A5*1/*1. The impact of CYP3A haplotypes on the metabolism of anti-malarial drugs in a large sample size cohort needs to be further evaluated.
